# Supplementary material for: Pten is necessary for the quiescence and maintenance of adult muscle stem cells
Source: Nat Commun. 2017 Jan 17;8:14328. doi: 10.1038/ncomms14328 (PMC5247606; doi:10.1038/ncomms14328)
Supplement: Supplementary Information — Supplementary Figures and Supplementary Tables [file ncomms14328-s1.pdf]

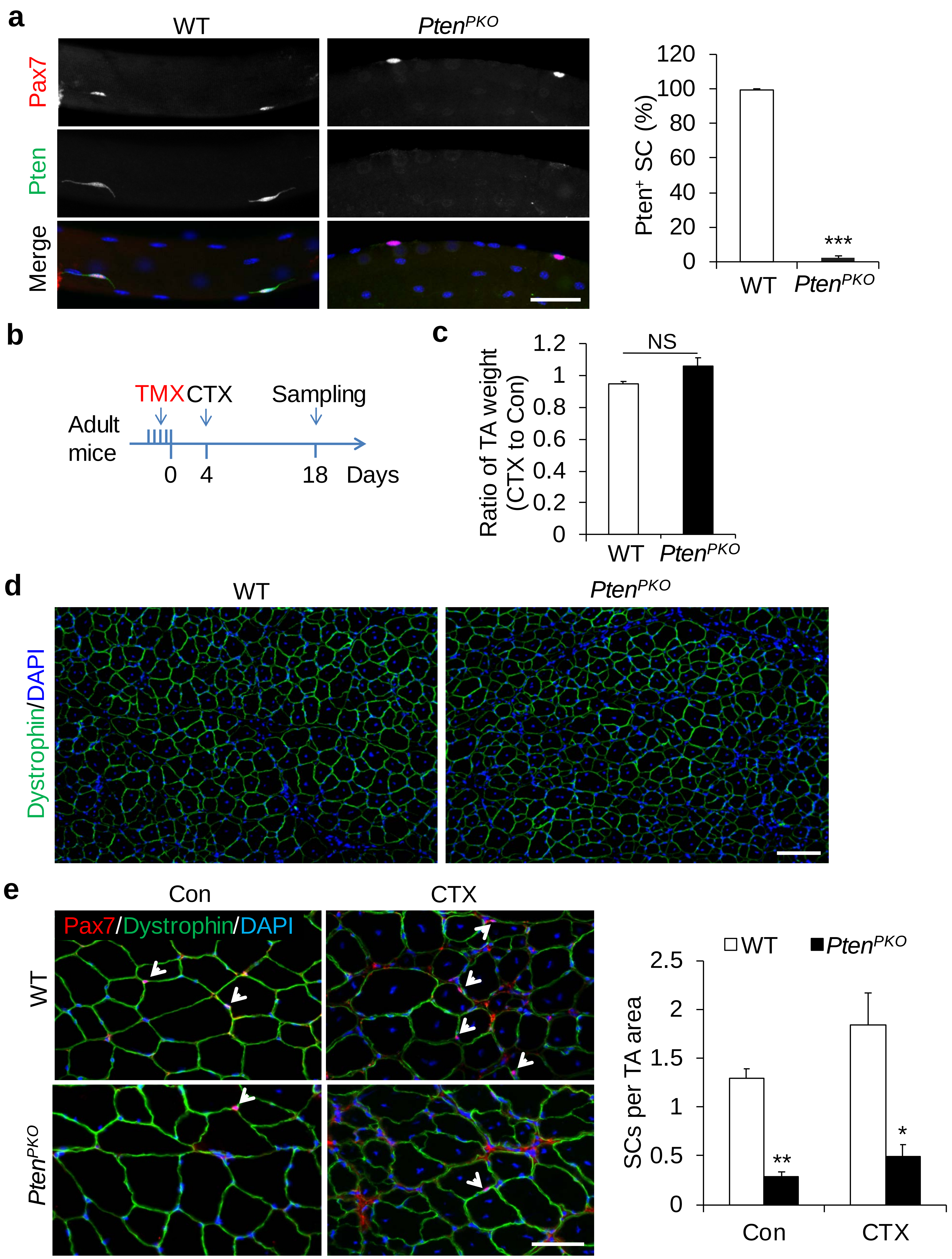

Supplementary Figure 1. Prior to depletion, *Pten* KO SCs are able to repair acute muscle injury, but depleted thereafter. (**a**) Left panel, immunofluorescence of Pax7 and Pten on fresh EDL myofibers isolated from WT and *Pten*^{PKO} mice 7 days after TMX induction. Scale bar, 50 µm. Right panel, quantification of the percentage of Pten^{+} SCs per EDL myofiber. Data represent mean ± s.e.m (*t*-test: ****P*<0.001; n=3, each group; 20 myofibers per animal). (**b**) Schematics showing TMX induction and CTX injection in adult WT and *Pten*^{PKO} mice. (**c**) Ratio of TA muscle weight after CTX injury. Data represent mean ± s.e.m (*t*-test: NS, *P*>0.05; n=4, each group). (**d**) Immunofluorescence of dystrophin in TA muscle cross-sections of WT and *Pten*^{PKO} mice. Scale bar, 100 µm. (**e**) Left panel, immunofluorescence of Pax7 and dystrophin on TA muscle cross-sections of WT and *Pten*^{PKO} mice. White arrow indicates SCs. Scale bar, 50 µm. Right panel, quantification of Pax7^{+} SC number. Data represent mean ± s.e.m (*t*-test: **P*<0.05; ***P*<0.01; n=4, each group).

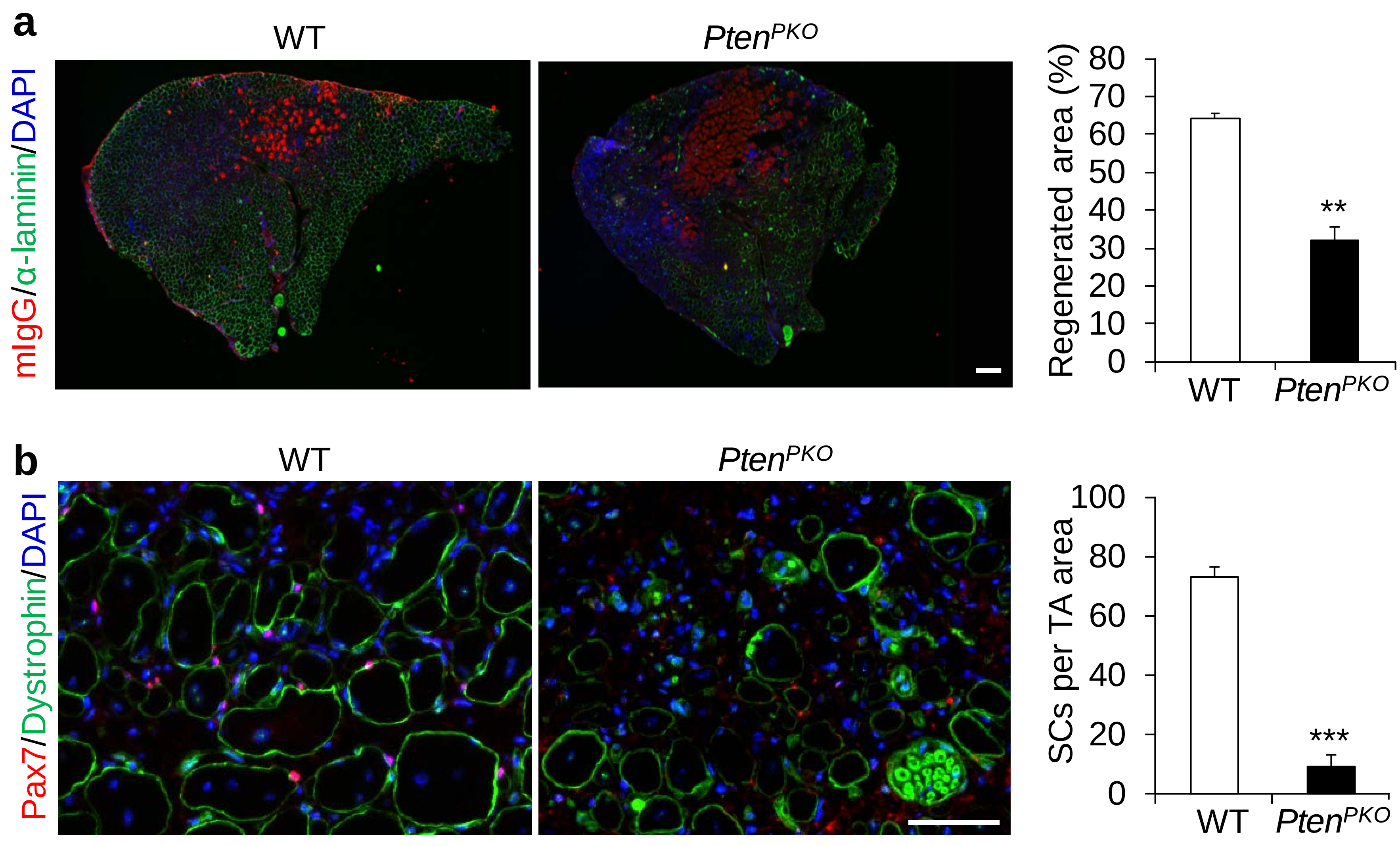

Supplementary Figure 2. Depletion of SCs impairs muscle regeneration in *Pten^PKO* mice. (**a**) Left panel, immunofluorescence of mIgG and α-laminin on TA muscle cross-sections 7 days after injury performed on Day 21 after TMX induction. Scale bar, 200 µm. Right panel, percentage of regenerated area on TA muscle cross-sections. Data represent mean ± s.e.m (*t*-test: ***P*<0.01; n=4, each group). (**b**) Left panel, immunofluorescence of Pax7 and dystrophin on TA muscle cross-sections 7 days after injury performed on Day 21 after TMX induction. Scale bar, 50 µm. Right panel, the number of SCs per TA area. Data represent mean ± s.e.m (*t*-test: ****P*<0.001; n=4, each group).

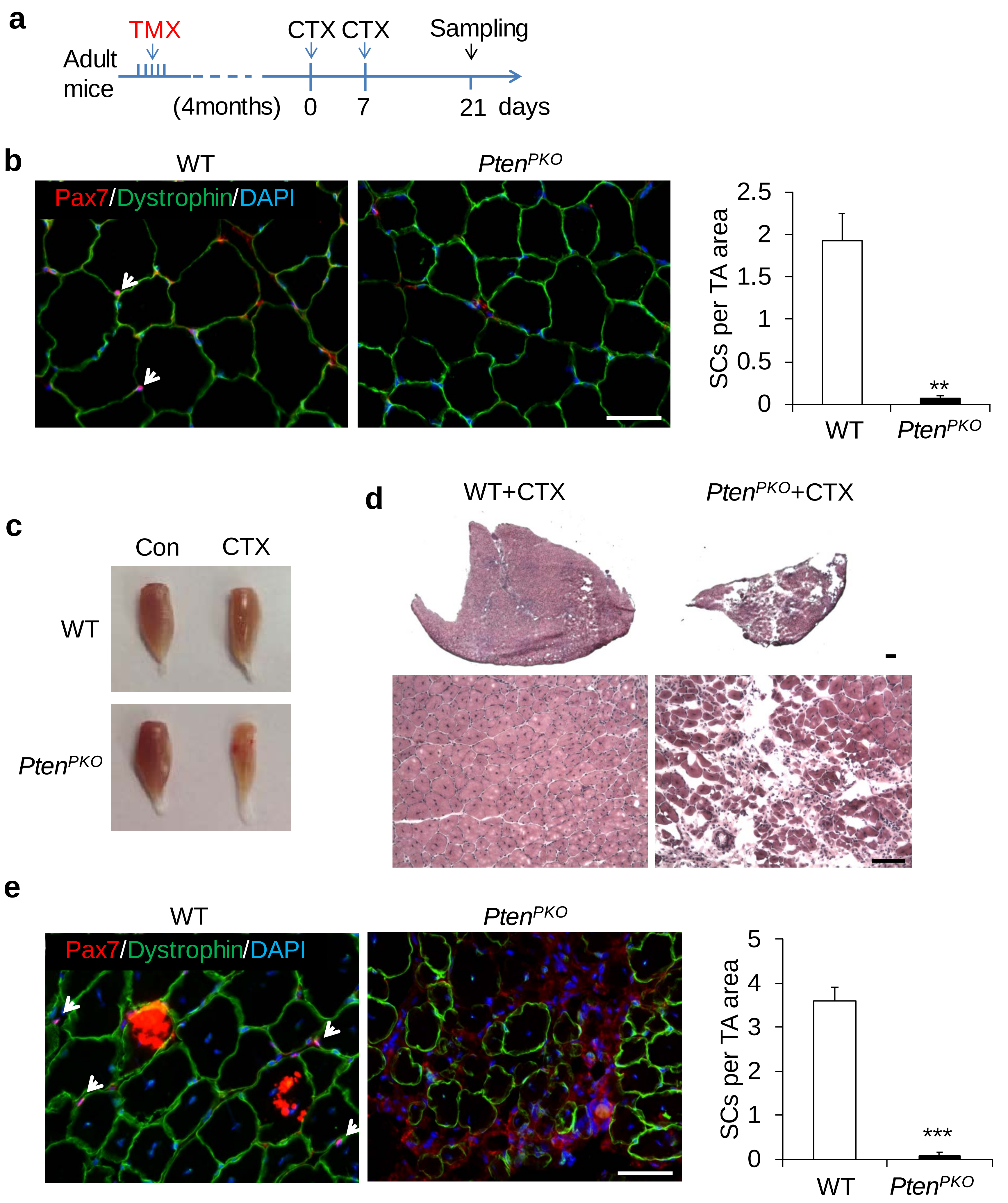

Supplementary Figure 3. The minority of remnant Pten⁺ SCs fail to repopulate SC pool and repairs muscle after long term TMX withdraw. (**a**) Schematics showing TMX induction and CTX injection in adult WT and *Pten^{PKO}* mice. (**b**) Left panel, immunofluorescence of Pax7 and dystrophin on uninjured TA muscle cross-sections of WT and *Pten^{PKO}* mice. White arrow indicates SCs. Scale bar, 50 µm. Right panel, quantification of Pax7^{+} SC number. Data represent mean ± s.e.m (*t*-test: ***P*<0.01; n=3, each group). (**c**) Representative images of TA muscles in WT and *Pten^{PKO}* mice. (**d**) H&E staining of TA muscle cross-sections (upper panels, scale bar: 200 µm) and magnified representative regenerated areas (bottom panels, scale bar: 50 µm. (**e**) Left panel, immunofluorescence of Pax7 and dystrophin on injured TA muscle cross-sections of WT and *Pten^{PKO}* mice. White arrow indicates SCs. Scale bar, 50 µm. Right panel, quantification of Pax7^{+} SC number. Data represent mean ± s.e.m (*t*-test: ****P*<0.001; n=3, each group).

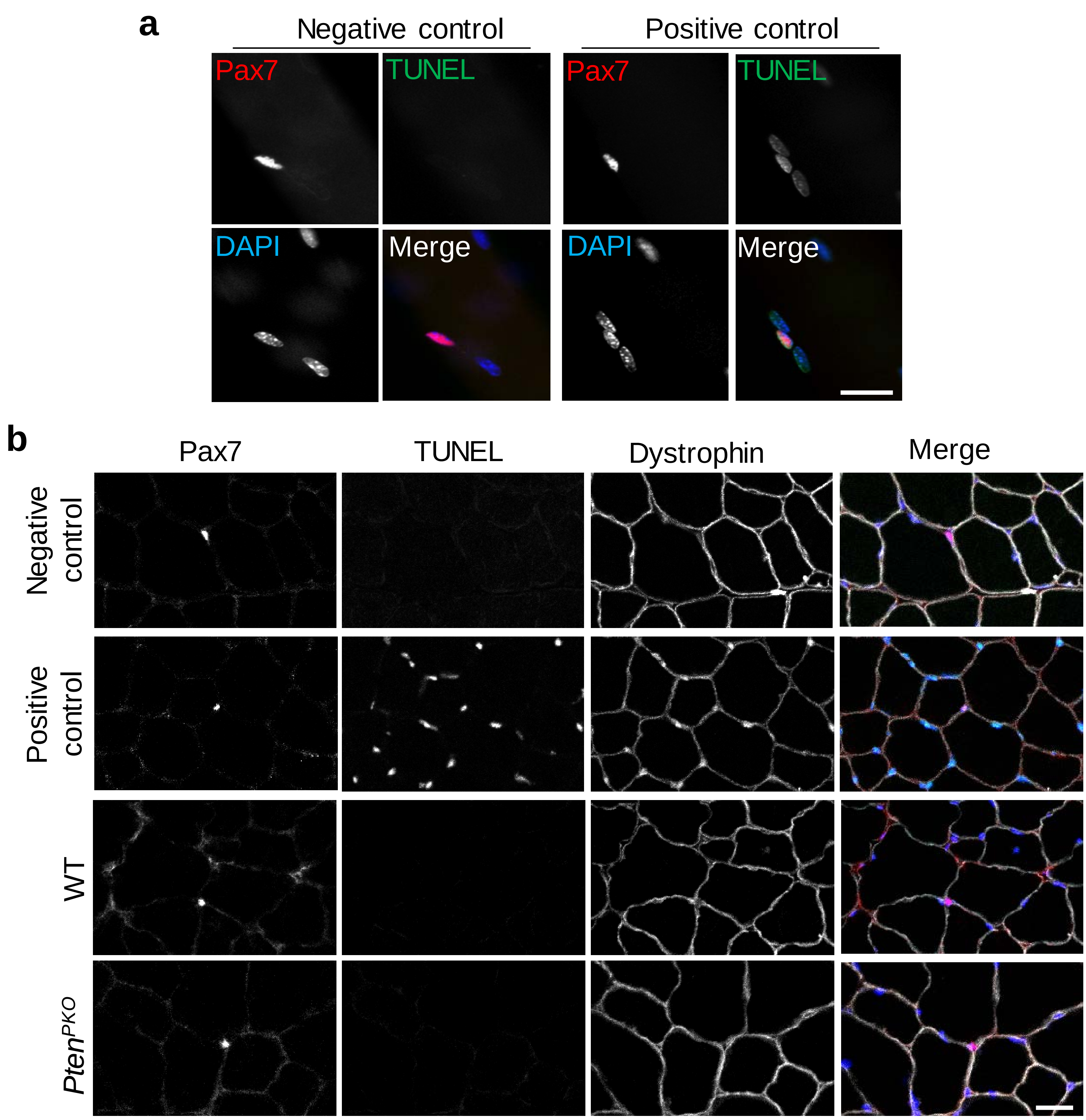

Supplementary Figure 4. *Pten* KO-induced SC depletion was not due to cell apoptosis. (**a**) Negative and positive control of immunofluorescence of TUNEL in SCs on fresh EDL myofibers isolated from WT mice. Scale bar, 20 µm. (**b**) Immunofluorescence of Pax7 (Red), TUNEL (Green) and dystrophin (White) on TA muscle cross-sections 7 days after TMX induction. Scale bar, 20 µm.

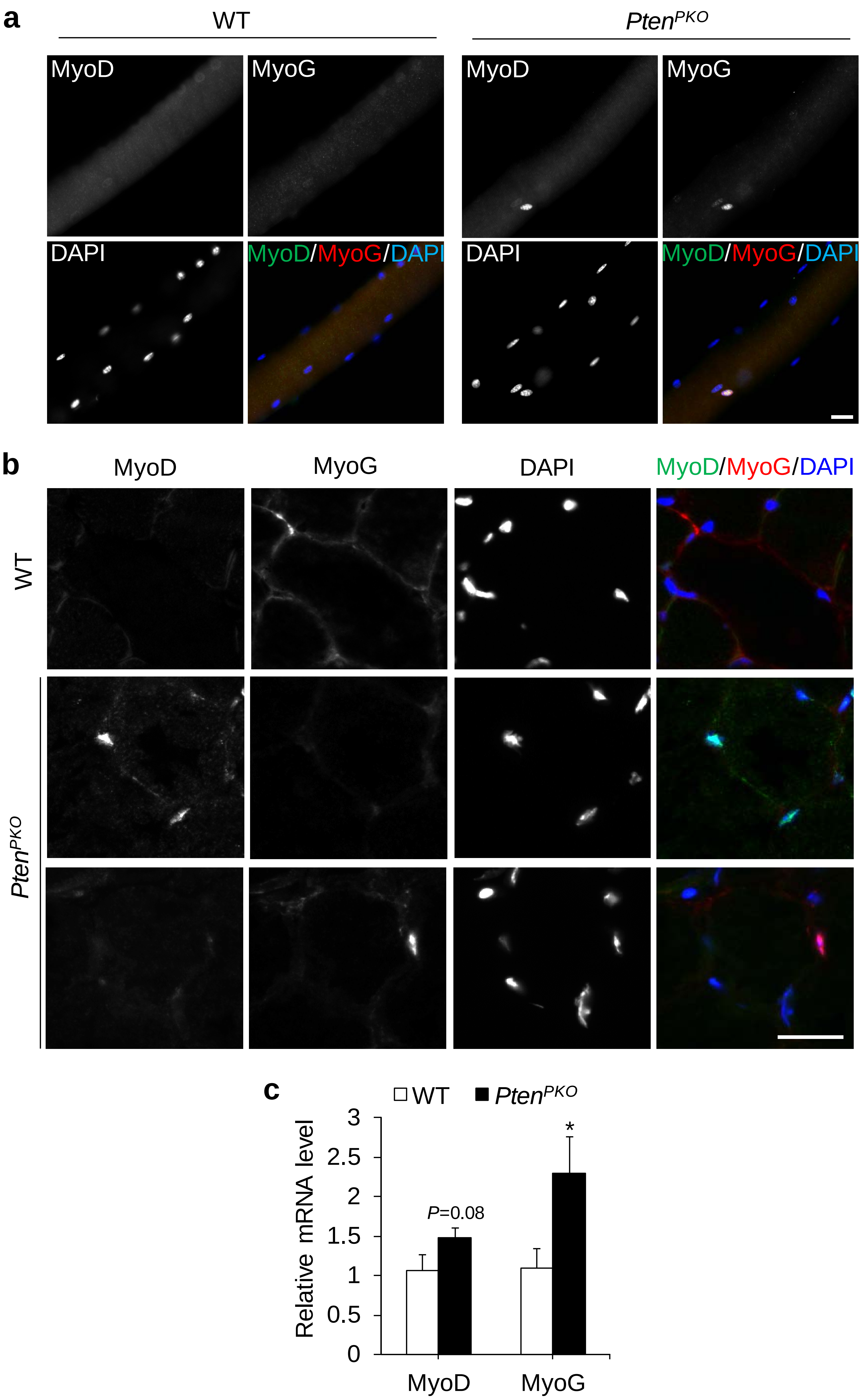

Supplementary Figure 5. *Pten* KO induces terminal differentiation of QSCs. (**a**) Immunofluorescence of MyoD and MyoG on freshly isolated EDL myofibers of WT and $Pten^{PKO}$ mice 4 or 9 days after TMX induction. Scale bar: 20 µm. (**b**) Immunofluorescence of MyoD and MyoG on TA muscle cross-sections of WT and $Pten^{PKO}$ mice 9 days after TMX induction. Scale bar, 50 µm. (**c**) qPCR showing relative mRNA levels of MyoD and MyoG in soleus muscles 9 days after TMX induction. Data represent mean ± s.e.m (*t*-test: *P<0.05; n=3, each group).

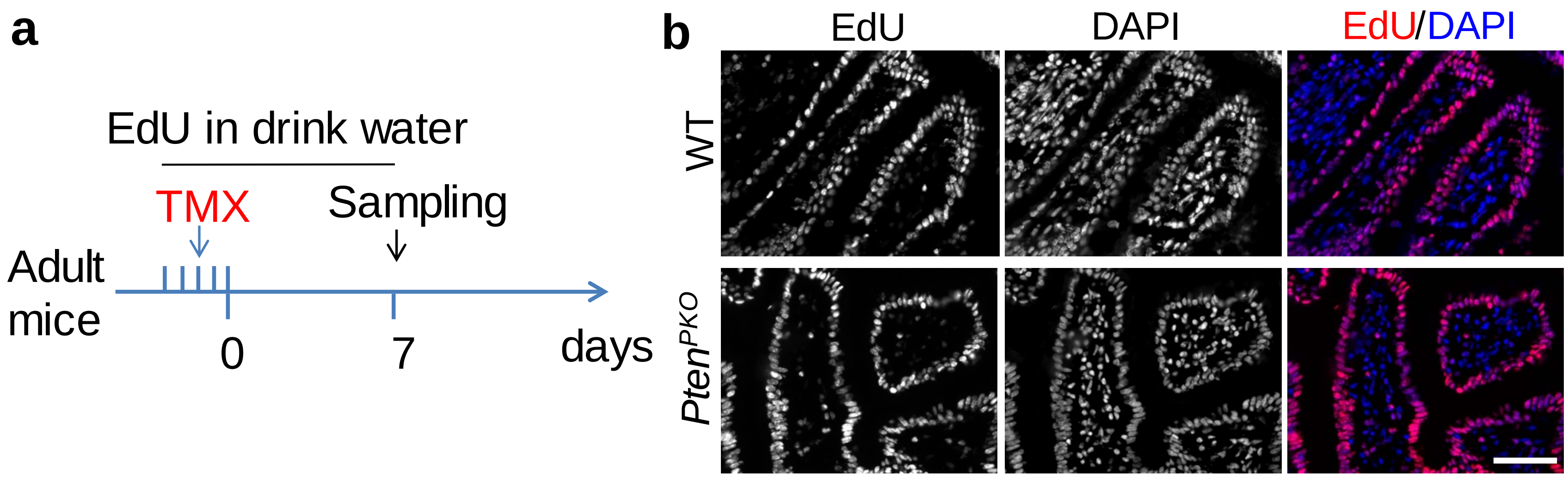

Supplementary Figure 6. Increased S-phase entry in *Pten*-null SCs. (**a**) Schematics showing TMX treatment and EdU injection in adult WT and *Pten*^{PKO} mice. (**b**) Positive control of EdU staining in intestine. Scale bar, 50 µm.

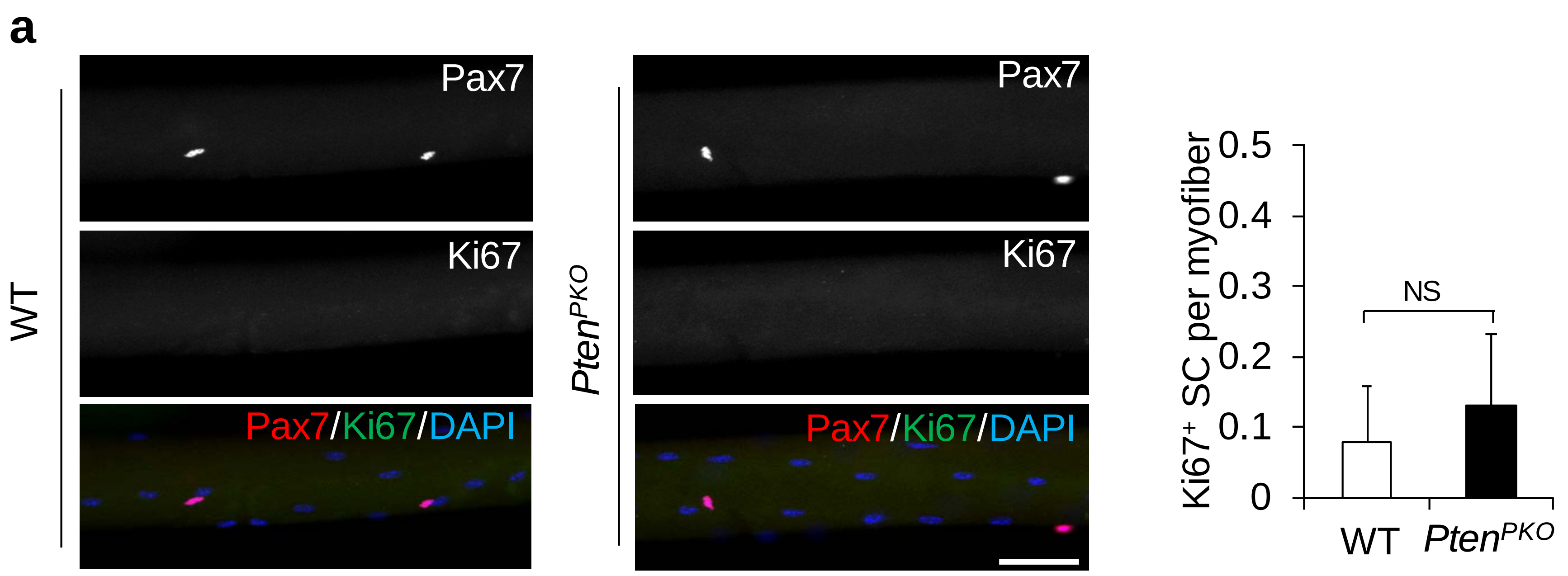

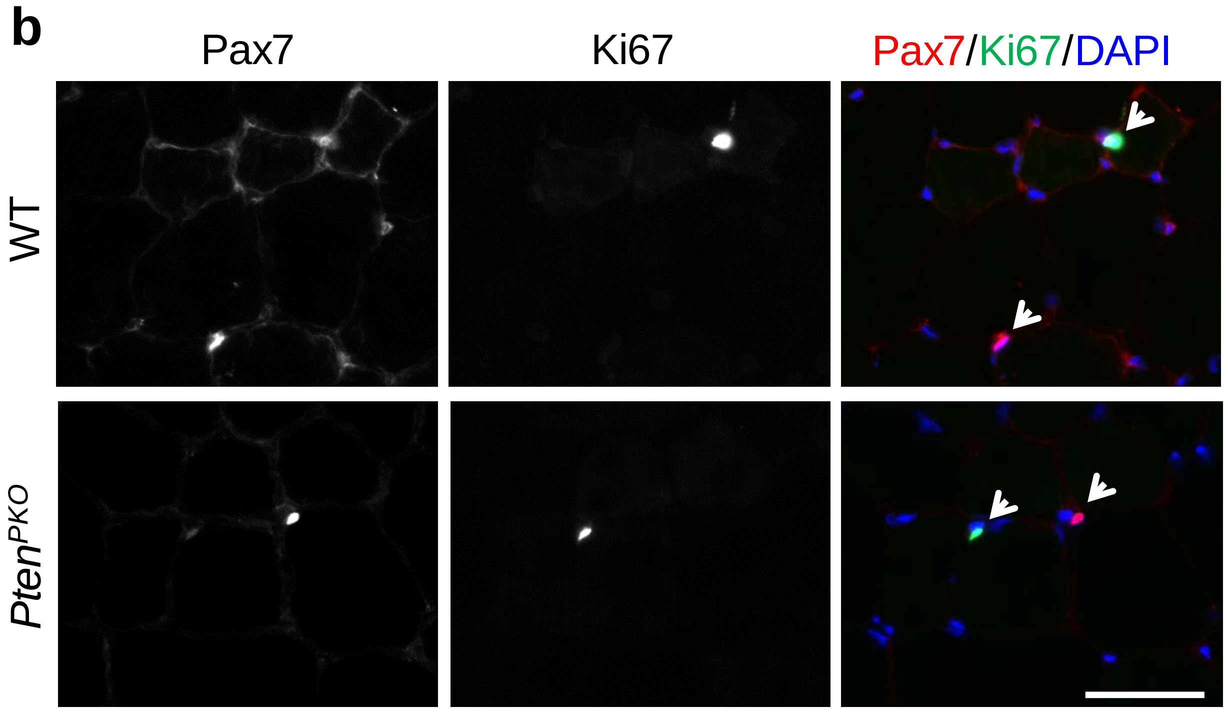

Ki67^{+} SCs

Number / Mouse	Total SCs	Ki67^{+} SCs
WT	53	3
Pten^{PKO}	22	3

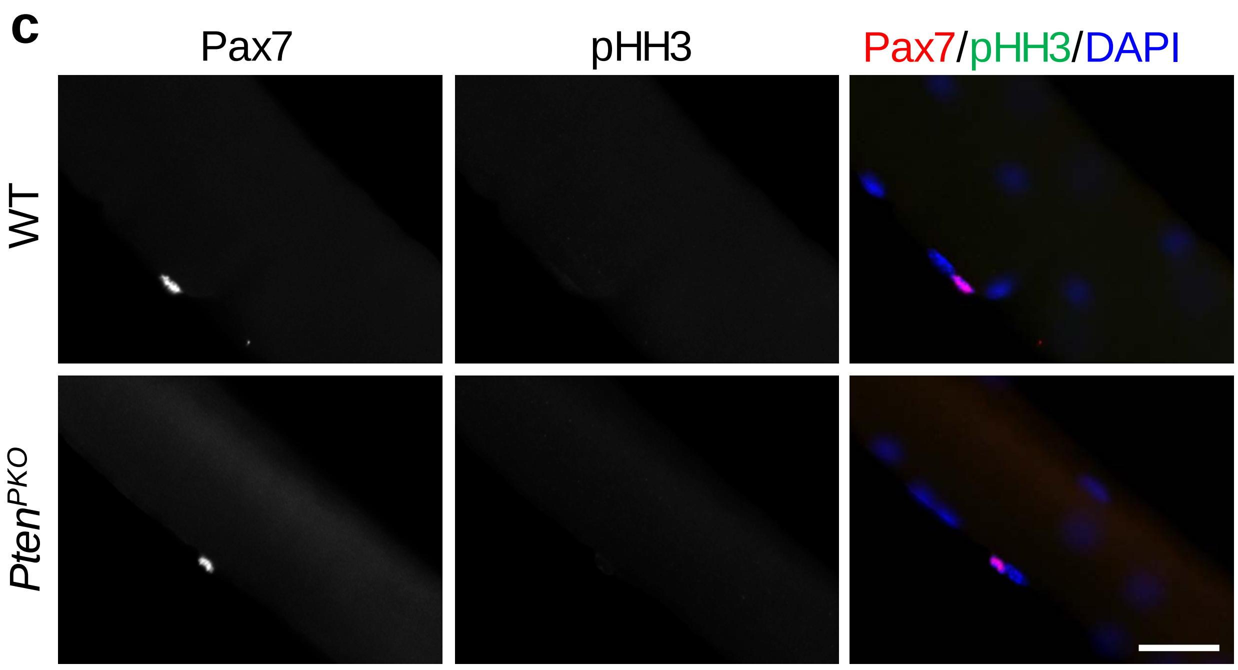

pHH3^{+} SCs

Number / Mouse	Total SCs	pHH3^{+} SCs
WT	444	0
Pten^{PKO}	392	0

Supplementary Figure 7. Majority of *Pten*-deficient SCs bypasses proliferation. (**a**) Left panel, immunofluorescence of Pax7 and Ki67 (mitotic marker) on fresh EDL myofibers isolated from WT and *Pten^{PKO}* mice 9 days after TMX induction. Scale bar, 50 µm. Right panel, quantification of the Ki67^{+} SCs on EDL myofibers. Data represent mean ± s.e.m (*t*-test: NS, P>0.05; n=3, each group; 20 myofibers per animal). (**b**) Left panel, immunofluorescence of Pax7 and Ki67 on TA muscle cross-sections 7 days after TMX induction. White arrow indicates SCs. Scale bar, 50 µm. Right panel, quantification of the Ki67^{+} SCs on TA muscle cross-sections. Total 53 SCs in WT and 22 SCs in *Pten^{PKO}* mice (n=3 animals, each group). (**c**) Left panel, immunofluorescence of Pax7 and Phospho-Histone H3 (pHH3, mitotic marker) on fresh EDL myofibers isolated from WT and *Pten^{PKO}* mice 7 days after TMX induction. Scale bar, 20 µm. Right panel, quantification of the pHH3^{+} SCs on EDL myofibers. Total 444 SCs in WT and 392 SCs in *Pten^{PKO}* mice (n=3 animals, each group).

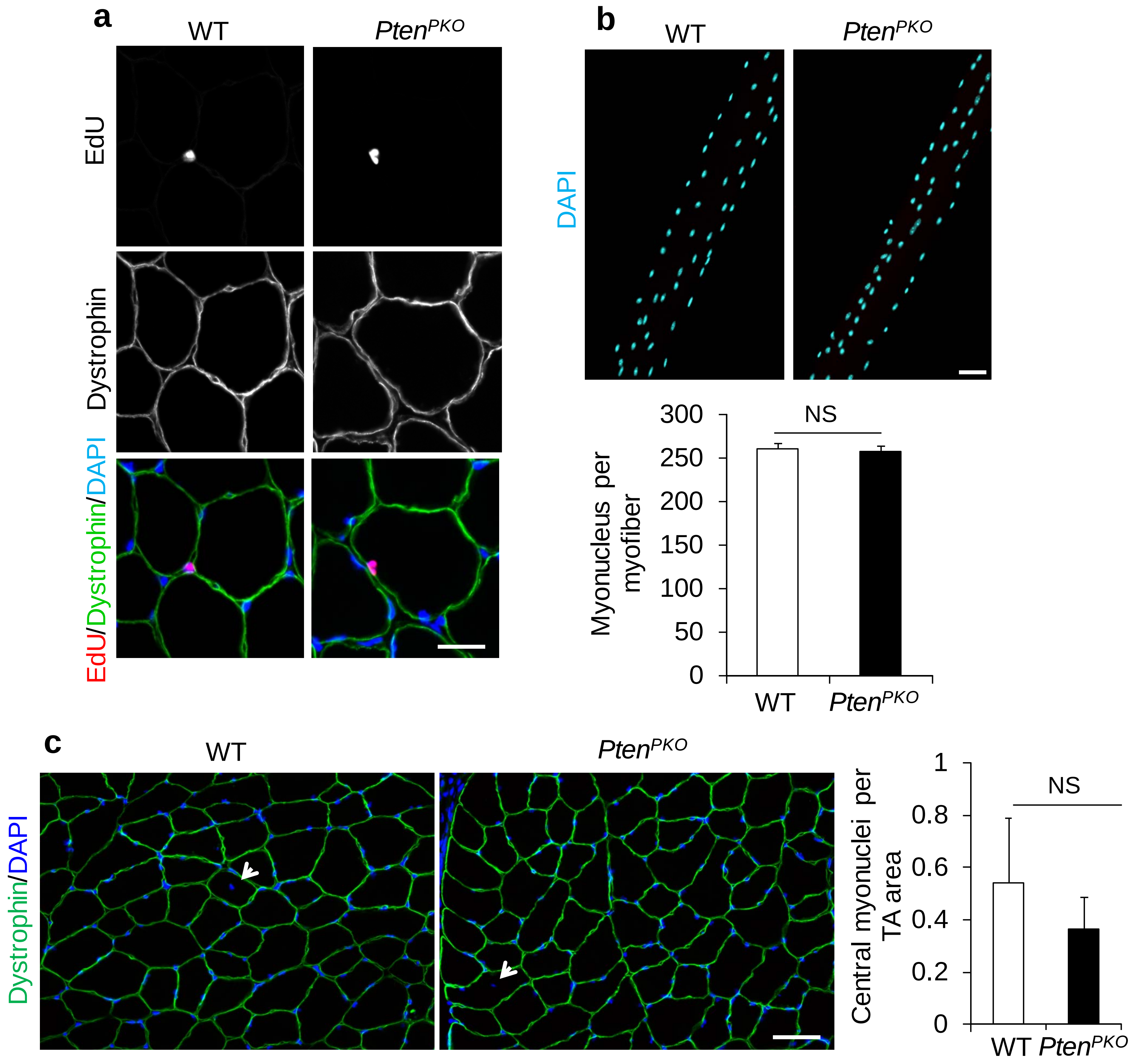

Supplementary Figure 8. Central myonuclei and total myonuclei number were not obvious changed in *Pten^{PKO}* mice. (**a**) Immunostaining of EdU and dystrophin on TA muscle cross-sections 7 days after TMX induction and EdU incoporation. Scale bar: 20 µm. (**b**) Upper panel, immunostaining of DAPI on fresh EDL myofibers isolated from WT and *Pten^{PKO}* mice 18 days after TMX induction. Scale bar: 50 µm. Bottom panel, quantification of the total number of myonuclei in myofibers. Data represent mean ± s.e.m (*t*-test: NS, *P*>0.05; n=4, each group, 25 myofibers per animal). (**b**) Left panel, immunofluorescence of dystrophin on TA muscle cross-sections in 7 days after TMX induction. White arrow indicates central myonuclei. Scale bar: 50 µm. Right panel, quantification of the number of central myonuclei in TA muscle cross-sections. Data represent mean ± s.e.m (*t*-test: NS, *P*>0.05; n=5, each group).

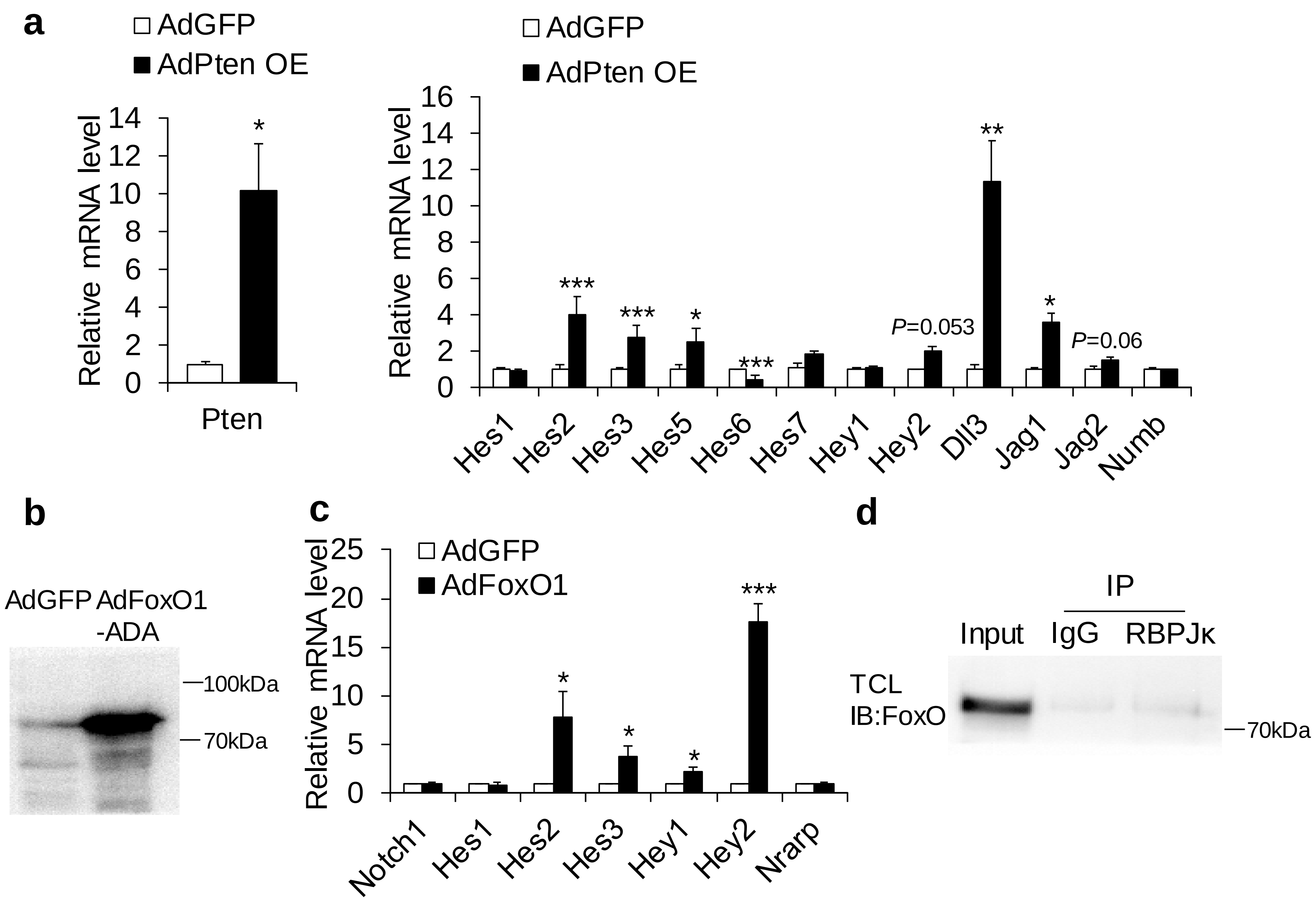

Supplementary Figure 9. Regulation of Notch downstream gene expression by Pten-Akt-FoxO1 pathway. (**a**) Relative mRNA levels of Pten (left) and Notch target genes (right) in primary myoblasts infected with AdGFP or AdPten adenovirus. Data represent mean ± s.e.m (*t*-test: *P<0.05; **P<0.01; ***P<0.001; n=3, each group). (**b**) Western blot analysis of FoxO1 expression in HEK293A cells infected with AdGFP or AdFoxO1-ADA adenovirus. (**c**) Relative mRNA levels of Notch target genes in primary myoblasts infected with AdGFP or AdFoxO1-ADA adenovirus. Data represent mean ± s.e.m (*t*-test: *P<0.05; ***P<0.001; n=6, each group). (**d**) Co-immunoprecipitation of endogenous FoxO1 and RBPJκ in primary myoblasts. TCL, total cellular lysate.

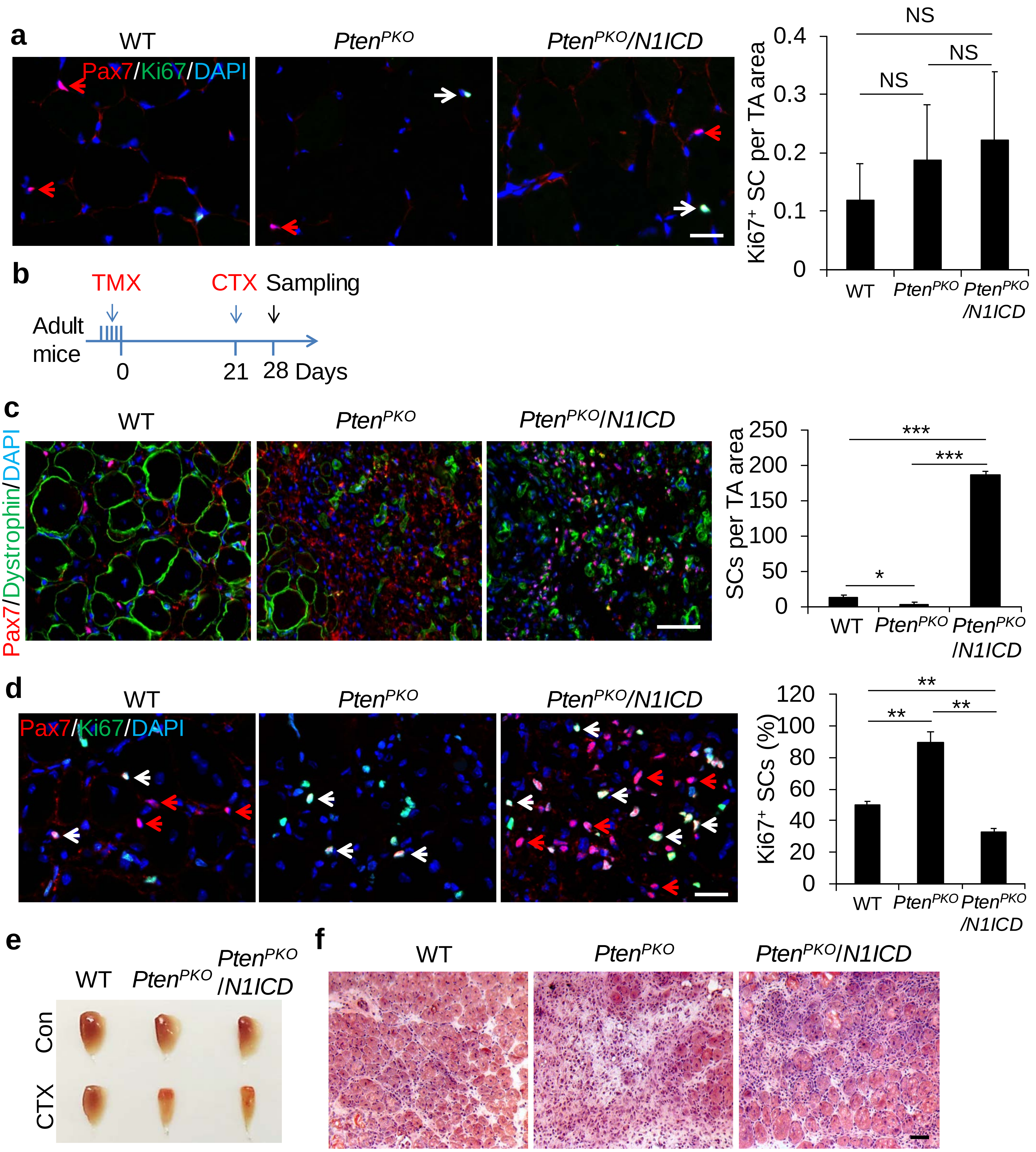

Supplementary Figure 10. Activation of Notch fails to rescue the regenerative defect induced by *Pten* deletion due to its inhibition on SC differentiation. (**a**) Left panel, immunofluorescence of Pax7 and Ki67 on TA muscle cross-sections of WT, *Pten^{PKO}* and *Pten^{PKO}/N1ICD* mice. Red arrow indicates Pax7$^+$Ki67$^-$ SCs, white arrow indicates Pax7$^+$Ki67$^+$ SCs. Scale bar, 50 µm. Right panel, quantification of Ki67$^+$ SC number. Data represent mean ± s.e.m (*t*-test: NS, *P*>0.05; n=4, each group). (**b**) Schematics showing TMX induction and CTX injection in adult WT, *Pten^{PKO}* and *Pten^{PKO}/N1ICD* mice. (**c**) Left panel, immunofluorescence of Pax7 and dystrophin on TA muscle cross-sections after injury. Scale bar, 50 µm. Right panel, quantification of SC number. Data represent mean ± s.e.m (*t*-test: **P*<0.05; ****P*<0.001; n=3, each group). (**d**) Left panel, immunofluorescence of Pax7 and Ki67 on TA muscle cross-sections after injury. Red arrow indicates Pax7$^+$Ki67$^-$ SCs, white arrow indicates Pax7$^+$Ki67$^+$ SCs. Scale bar, 50 µm. Right panel, quantification of the percentage of Ki67$^+$ SC number. Data represent mean ± s.e.m (*t*-test: **P*<0.01; n=4, each group). (**e**) Representative images of TA muscles after injury. (**f**) H&E staining of TA muscle cross-sections. Scale bar: 50 µm).

Fig. 1c

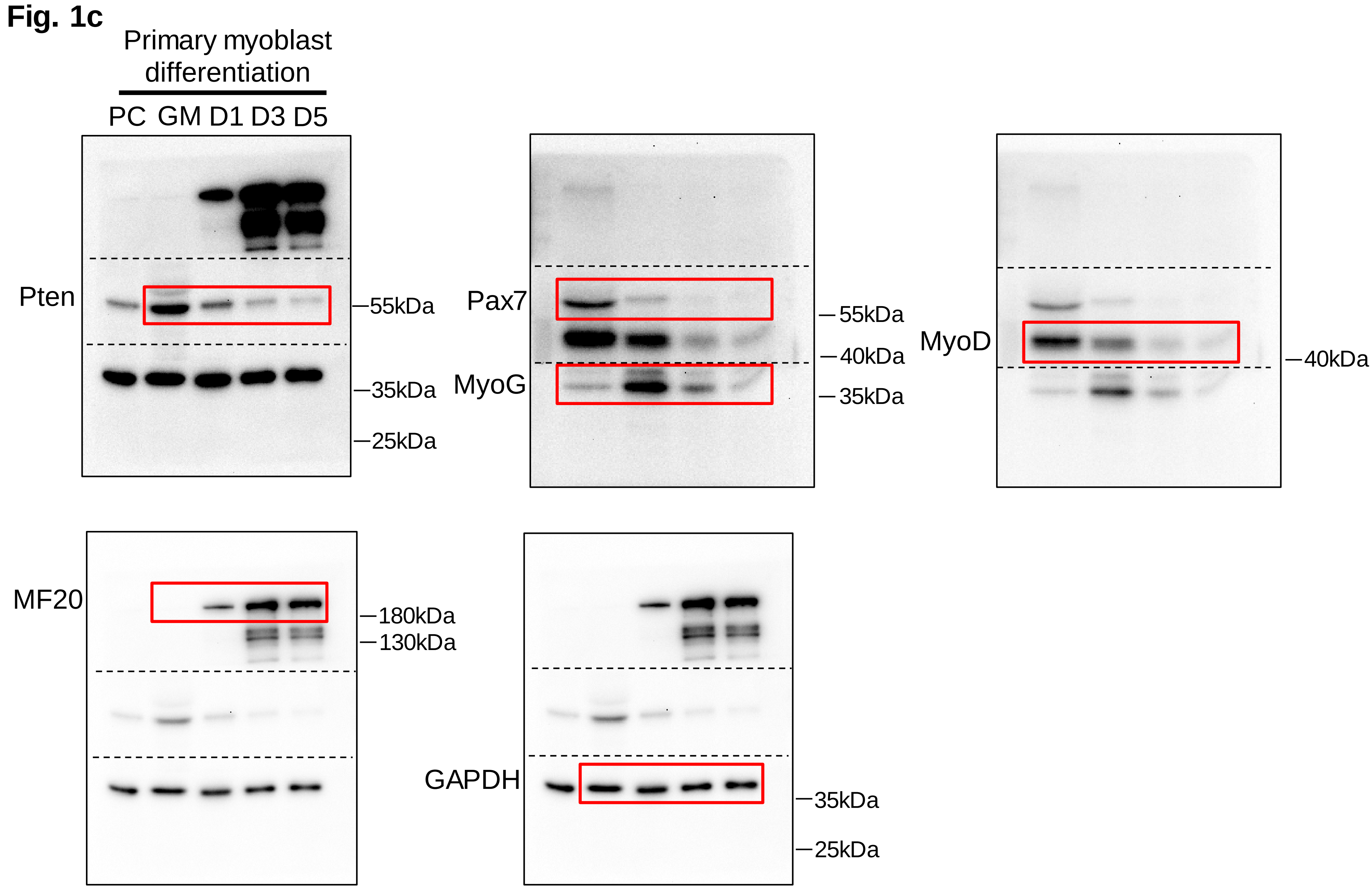

Fig. 7e

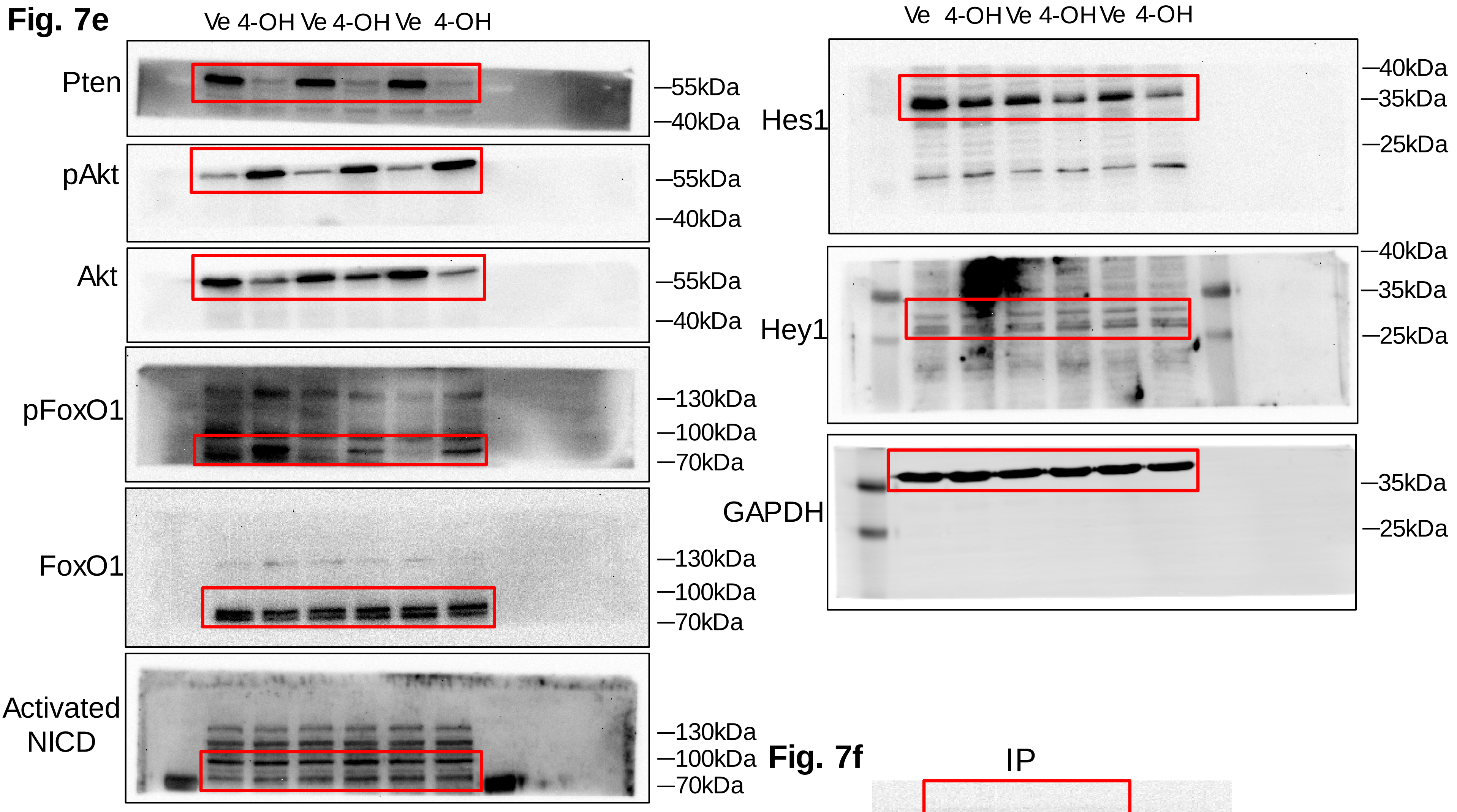

Fig. 7f

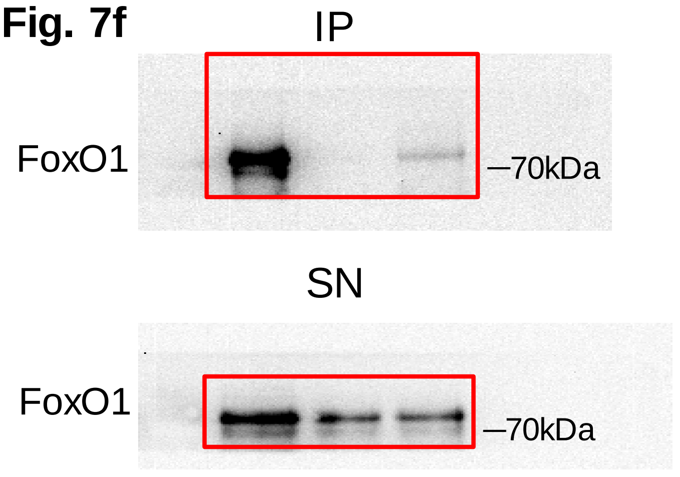

Supplementary Figure 11. Uncropped original blots in main figures. PC: Positive control sample.

Supplementary Table 1

List of antibodies used for immunofluorescence in this study

Antibody	Company and Catalog number	Host	Dilution factor
Pax7 (PAX7)	Developmental Studies Hybridoma Bank	Mouse	1:10
Pten	Cell Signaling, #9188	Rabbit	1:300
MyoD	Santa Cruz Biotechnology, sc-760	Rabbit	1:300
MyoG (F5D)	Developmental Studies Hybridoma Bank	Mouse	1:500
MyHC (MF20)	Developmental Studies Hybridoma Bank	Mouse	1:50
Laminin A	AbCam, #ab26300	Rabbit	1:1000
Dystrophin	AbCam, #ab15277	Rabbit	1:1000
Ki67	AbCam, #ab15580	Rabbit	1:1000
phospho-Ser473 Akt	Cell Signaling, #4060	Rabbit	1:500
phospho-S235/6 S6	Cell Signaling, #2211	Rabbit	1:500
FoxO1	Cell Signaling, #2880	Rabbit	1:500
phospho-Histone H3	Cell Signaling, #9701	Rabbit	1:1000
Cleaved-Caspase3	Cell Signaling, #9661	Rabbit	1:500
Alexa568 goat anti-mouse IgG1	Invitrogen, #A-21124	Goat	1:1000
Alexa488 goat anti-mouse IgG1	Invitrogen, #A-21121	Goat	1:1000
Alexa488 goat anti-rabbit IgG	Invitrogen, #A-11034	Goat	1:1000
Alexa647 goat anti-mouse IgG2b	Invitrogen, #A-21242	Goat	1:1000
Alexa647 goat anti-rabbit IgG	Invitrogen, #A-21244	Goat	1:1000

Supplementary Table 2

List of primers used for qRT-PCR in this study

Gene name	Primer sequence (5'-3')
18s	F:AGT CCC TGC CCT TTG TAC ACA<br>R:CGA TCC GAG GGC CTC ACT A
Pten	F:GGA AAG GGA CGG ACT GGT GTA A<br>R:GCA GTG CCA CGG GTC TGT AAT C
MyoD	F:GGC TAC GAC ACC GCC TAC TA<br>R:CGA CTC TGG TGG TGC ATC TG
MyoG	F:TGC CCA GTG AAT GCA ACT CC<br>R:TTG GGC ATG GTT TCG TCT GG
Hes1	F:GCA CAG AAA GTC ATC AAA GCC<br>R:TTG ATC TGG GTC ATG CAG TTG
Hes2	F:ACA ATT ACC CTG GGC ACG CTA C<br>R:CCT GTA GCC TGG AGC ATC TTC AAA
Hes3	F:GCA CGC ATC AAC GTG TCA C<br>R:TGA GTT CTG GAG GCT TCT CAT
Hes5	F:ATG CTC AGT CCC AAG GAG AA<br>R:TAG TCC TGG TGC AGG CTC TT
Hes6	F:GCC GGA TTT GGT GTC TAC AT<br>R:TCC TGA GCT GTC TCC ACC TT
Hes7	F:CGG GAG CGA GCT GAG AAT AG<br>R:CAC GGC GAA CTC CAG TAT CT
Hey1	F:TGA ATC CAG ATG ACC AGC TAC TGT<br>R:TAC TTT CAGACT CCG ATC GCT TAC
Hey2	F: AAG CGC CCT TGT GAG GAA AC<br>R:GGT AGT TGT CGG TGA ATT GGA C
HeyL	F:CAG ATG CAA GCC CGG AAG AA<br>R:ACC AGA GGC ATG GAG CAT CT
Dll1	F:GCA GGA CCT TCT TTC GCG TAT<br>R:AAG GGG AAT CGG ATG GGG TT
Dll3	F:CTG GTG TCT TCG AGC TAC AAA T<br>R:TGC TCC GTA TAG ACC GGG AC
Jag1	F:CCT CGG GTC AGT TTG AGC TG<br>R:CCT TGA GGC ACA CTT TGA AGT A
Jag2	F:CAA TGA CAC CAC TCC AGA TGA G<br>R:GGC CAA AGA AGT CGT TGC G
Numb	F:CTT CCC AGT TAA GTA CCT CGG C<br>R:CCC GTT TTT CCA AAG AAG CCT

Supplementary Table 3

List of antibodies used for western blot analysis in this study

Antibody	Company and Catalog number	Host	Dilution factor
Pax7 (PAX7)	Developmental Studies Hybridoma Bank	Mouse	1:50
Pten	Cell Signaling, #9188	Rabbit	1:2000
MyoD	Santa Cruz Biotechnology, sc-760	Rabbit	1:500
MyoG (F5D)	Developmental Studies Hybridoma Bank	Mouse	1:1000
MyHC (MF20)	Developmental Studies Hybridoma Bank	Mouse	1:100
Activated Notch1	AbCam, #ab8925	Rabbit	1:1000
Hes1	Santa Cruz Biotechnology, sc-25392	Rabbit	1:500
HRT1 (Hey1)	Santa Cruz Biotechnology, sc-28746	Rabbit	1:500
phospho-Ser473 Akt	Cell Signaling, #4060	Rabbit	1:3000
Akt (pan)	Cell Signaling, #4691	Rabbit	1:3000
phospho-FoxO1-Thr24	Cell Signaling, #9464	Rabbit	1:2000
FoxO1	Cell Signaling, #2880	Rabbit	1:2000
GAPDH (6C5)	Santa Cruz Biotechnology, sc-32233	Mouse	1:3000
HRP-conjugated goat anti-mouse IgG	Jackson ImmunoResearch , #115-035-003	Goat	1:10,000
HRP-conjugated goat anti-rabbit IgG	Jackson ImmunoResearch , #111-035-003	Goat	1:10,000
